# Cluster-based psychological phenotyping and differences in anxiety treatment outcomes

**DOI:** 10.1038/s41598-023-28660-7

**Published:** 2023-02-21

**Authors:** Veronique A. Taylor, Alexandra Roy, Judson A. Brewer

**Affiliations:** 1 Mindfulness Center, Brown University School of Public Health, 121 South Main Street, Providence, RI 02903 USA; 2 Department of Psychiatry and Human Behavior, Warren Alpert Medical School of Brown University, Providence, RI USA

**Keywords:** Psychology, Human behaviour

## Abstract

The identification of markers of mental health illness treatment response and susceptibility using personalized medicine has been elusive. In the context of psychological treatment for anxiety, we conducted two studies to identify psychological phenotypes with distinct characteristics related to: psychological intervention modalities (mindfulness training/awareness), mechanism of action (worry), and clinical outcome (generalized anxiety disorder scale scores). We also examined whether phenotype membership interacted with treatment response (Study 1) and mental health illness diagnosis (Studies 1–2). Interoceptive awareness, emotional reactivity, worry, and anxiety were assessed at baseline in treatment-seeking individuals (Study 1, n = 63) and from the general population (Study 2, n = 14,010). In Study 1, participants were randomly assigned to an app-delivered mindfulness program for anxiety for two months or treatment as usual. Changes in anxiety were assessed 1 and 2 months post-treatment initiation. In studies 1–2, three phenotypes were identified: ‘severely anxious with body/emotional awareness’ (cluster 1), ‘body/emotionally unaware’ (cluster 2), and ‘non-reactive and aware’ (cluster 3). Study 1’s results revealed a significant treatment response relative to controls (ps < 0.001) for clusters 1 and 3, but not for cluster 2. Chi-square analyses revealed that phenotypes exhibited significantly different proportions of participants with mental health diagnoses (studies 1–2). These results suggest that psychological phenotyping can bring the application of personalized medicine into clinical settings.

Registry name and URL: Developing a novel digital therapeutic for the treatment of generalized anxiety disorder https://clinicaltrials.gov/ct2/show/NCT03683472?term=judson+brewer&draw=1&rank=1.

*Trial registration*: Registered at clinicaltrials.gov (NCT03683472) on 25/09/2018.

## Introduction

The goal of personalizing medicine—matching treatment at the level of the individual—to facilitate the diagnosis and treatment of diseases has challenged researchers and clinicians for decades^1^. This approach is based on the notion that inter-individual variability in various attributes (e.g. genetic, brain and physiological function, environmental exposure, behavioral and personality profile) is associated with heterogeneity in disease processes (disease progression, underlying factors/mechanisms) and treatment responses^1^. Personalized medicine began showing promise from genetic studies in which responses to drug treatments differed depending on individual’s specific genetic profile^2–4^. However, with respect to personalizing medicine in mental health (e.g. psychiatry), there is a need for more research directed toward personalizing *psychological* interventions to optimize treatment selection and efficacy for individual patients^5^. Ideal personalized medicine approaches would be low cost, easily implemented and scalable at a population level.

Particularly with respect to anxiety disorders, there is a need for establishing robust markers of illness susceptibility and treatment responses so that these findings can be applied to key clinical decision making processes (e.g. optimal treatment selection for individual patients at their first clinic visit)^6^. Individuals with anxiety disorders, such as generalized anxiety disorder (GAD), display varying responses to pharmacological agents and psychotherapeutic approaches: response rates to anti-anxiety medications vary from 30 to 68%^7^, while 46% of patients showed clinical improvement from psychotherapeutic treatment^8^. In addition, other recent reviews of the literature discuss variable response rates to different pharmacological agents and/or psychological treatment for anxiety disorders^9,10^. A recent meta-analysis also showed individual variability to psychological anxiety treatment outcomes, such that post-treatment symptom alleviation was significantly enhanced for patients exhibiting early response to treatment (in the first 4 weeks) vs those not showing such early improvements^11^.

There is currently no systematic process for the selection of anxiety treatments on the basis of empirically-supported markers of intervention success^6^. In clinical practice, treatment selection is guided by factors such as cost/benefit evaluation of clinical vs. side effects, or personal experience of the healthcare practitioner^6^.

Methods to identify subgroups with shared characteristics that are associated with treatment outcomes are being explored^12^. For example, unsupervised machine learning methods can be used to detect clusters of participants that share similar patterns or combinations of various characteristics^13^. Subgroups sharing common patterns of features can then be compared based on their treatment outcomes. However, studies including machine learning approaches to identifying markers that relate to psychological clinical treatment responses are scarce but proof-of-concept reports are emerging^14^.

A recent study used clustering methods to determine phenotypes of depression treatment resistance based on the most salient self-reported clinical features (e.g. socio-demographic characteristics, symptom severity), with models showing acceptable ranges of predictive accuracy over treatment outcomes^14^. These results suggest that “psychological phenotyping” may be a useful step forward in the implementation of precision medicine, and have the advantages of low-cost/high accessibility to researchers and clinicians as compared to genetic or neuroimaging biomarkers. The identification of psychological phenotypes could also guide prospective investigations of underlying genetic variants or neurobiological processes involved to uncover bio-/neuromarkers. Self-reported psychological assessments can also be rapidly collected and analyzed in clinical settings, thereby rendering them easily implementable in clinical practice, and scalable such that access can be provided at the population level.

Three key components may be needed to optimally determine an individual’s response to a psychological intervention: (1) traits/skills the intervention is aimed at developing, (2) mechanistic factors that are related to the disease process, and (3) clinical outcomes.

With respect to treatment-related characteristics, mindfulness training (MT) is increasingly being studied as a treatment for several mental health disorders^15,16^. Mindfulness-based approaches are aimed at developing awareness of present-moment experiences with acceptance^17^. Such approaches foster non-reactivity or ‘being with’ unpleasant states, rather than reacting by carrying out a behavior to distract from or avoid such states (e.g. smoking, emotional eating, worrying)^18^, with demonstrated efficacy on reducing anxiety symptoms. However, many of these effects are based on studies that do not have appropriate control groups, warranting an important need for further study [for reviews, see^15,16^].

To more precisely identify psychological markers relevant to treatment outcomes, it would be informative to not only involve characteristics relevant to the disordered process under study—anxiety–, but also to mechanistic aspects involved in its formation/maintenance and that are targeted by the psychological intervention. Thus, one mechanism identified in developing/maintaining anxious symptoms is the particular way people respond, manage, or relate to their anxiety^19^. As such, worry, a central feature of anxiety^20^, can be described as maintained by the relief it provides in avoiding deep-seated core emotions that could be perceived as threatening^19^. This immediate benefit from experiential avoidance may reinforce and maintain further engagement in worry^19^. To disentangle the maladaptive cycle of worry and anxiety, Unwinding Anxiety (UA) is a mindfulness training program aimed at building up skills to develop awareness of these processes. In developing more mindful awareness to these processes, people can learn to be and work with unpleasant states^18,21,22^, rather than avoid or react to them and perpetuate the habitual cycle of worry^15,16^. A previous study has shown that MT for anxiety’s reductions in GAD-7 scores were mediated by reductions in worry, in support of worry reduction as a mechanistic target for the impact of MT training on anxiety.

Recently, app-delivered MT has shown preliminary efficacy in symptom reduction: single-arm trials with anxious physicians^23^ and a randomized controlled trial from our laboratory conducted by Roy et al. which included individuals with GAD showed 57% and 67% reductions in clinical symptoms of anxiety respectively (Generalized Anxiety Disorder-7 scores, GAD-7)^24^. In the latter study, 36% of subjects in the experimental group did not achieve remission, suggesting the presence of inter-individual variability in treatment outcomes. Identifying baseline characteristics of participants who do not respond as robustly to treatment could help match individuals to MT vs. other therapeutic modalities –saving time and cost, while potentially improving outcomes.

To date, neuroimaging, genetic, and behavioral findings on anxiety disorders have not yet translated into robust and established markers of anxiety and its treatment outcomes to guide clinical recommendations^6^. Here, we conducted two studies, in which we adopted a data-driven, unsupervised learning approach to identify psychological phenotypes that may interact with anxiety treatment matching and outcomes. In Study 1, we used a clustering approach to detect the presence of subgroups of participants with shared psychological attributes and whether group membership could interact with treatment outcomes in a randomized controlled trial (N = 63). We included measures that captured core aspects of treatment (non-reactivity and interoceptive awareness)^25,26^, mechanism (Penn-State Worry Questionnaire)^20,27^, and clinically-relevant outcomes (GAD-7)^28^. In Study 2, we examined whether the psychological phenotypic clusters that were discovered in Study 1 could be identified in the general population (N = 14,010). We then examined whether sub-group membership could be associated with mental health diagnoses (Studies 1 and 2).

## Study 1: methods and materials

The overall effects of app-based MT for anxiety on anxiety outcomes have been reported elsewhere (NCT03683472 registered at clinicaltrials.gov on 25/09/2018^24^). The trial protocol has not been previously published. These results^24^ are distinct from those presented here, as the present results report on the impact of cluster membership on responses to anxiety treatment.

### Participants

The 63 participants (see Supplementary Materials for a description of recruitment procedures, participant flow, and inclusion criteria) enrolled in the study were, on average 43 (± 15) years of age for the treatment as usual combined with mindfulness training (TAU + MT) group, and 41 (± 16) years of age for the treatment as usual (TAU) group. The TAU + MT group consisted of 28 females and two males, while the TAU group consisted of 29 females, three males, and one individual who selected other. These and other participant characteristics are displayed in Table S3 (Supplementary Materials). All participants provided written informed consent. This study was granted ethical approval by the Brown University Institutional Review Board and was performed in accordance with the relevant guidelines/regulations (i.e. the principles of the Declaration of Helsinki).

### Intervention

The app-delivered MT program (Unwinding Anxiety, ‘UA’) which was used in this study as well as the experimental procedure are fully described in the Supplementary Materials section.

### Measures

Moreover, scores on self-report questionnaires (*Generalized Anxiety Disorder 7-item (GAD-7), Penn State Worry Questionnaire (PSWQ),* Non-Reactivity Subscale of the Five Facet Mindfulness Questionnaire (FFMQ)), and the Multidimensional Assessment of Interoceptive Awareness (MAIA) were assessed at baseline, one, and two months after initiation of the intervention (see Supplementary Materials section for full description).

### Data analysis

#### Cluster analyses

A total of 63 participants were included in these analyses. Following previous recommendations^29^, we first used a hierarchical agglomerative method (after having reduced the data using principal component analysis (PCA)) in order to determine the number of clusters present within the data. This was followed by an iterative partitioning approach (k-means) to minimize within-cluster distances and maximize between-cluster distances (see the Supplementary Materials Section for full cluster analyses procedure description).

#### Interaction between subgroup membership on responses to MT

Sixty-one participants were included in these analyses due to two participants not completing follow-up assessment surveys. To determine whether psychological phenotype significantly interacted with treatment responses to MT, a mixed measures 3-way ANOVA using TIME (baseline, 1, 2 months post-intervention) as a repeated measures factor, as well as GROUP (TAU + MT, TAU) and CLUSTER as between-subjects factors was conducted on GAD-7 scores. Follow-up analysis (within cluster GROUP X TIME mixed measures ANOVA and contrasts) were conducted to break down a TIME X GROUP X CLUSTER interaction effect, where applicable.

To examine whether participants in different clusters may have exhibited different treatment responses due to reduced levels of engagement in the intervention, a TIME X CLUSTER ANOVA was conducted on engagement measures in the TAU + MT participants, for which levels of TIME included were one and two months post-intervention (since no module had been completed at baseline). These data analyses are described in fuller detail in the Supplementary Materials section.

#### Association between cluster membership and anxiety disorder or depression diagnosis

To determine the presence of an association between cluster membership and anxiety disorder or depression diagnosis (panic disorder, OCD, GAD, social anxiety disorder, PTSD, agoraphobia, depression), we conducted chi-square analyses using a separate model for each type of diagnosis and a Bonferroni—corrected p value of 0.007 to account for multiple tests.

## Study 2: methods and materials

### Participants

The sample consisted of 14,260 participants. Potential participants were recruited by Sharecare Inc., utilizing Sharecare’s QualityHealth recruitment funnel which individuals opt into based on health conditions that are of interest to them. Email recruitment invitations were sent to those indicating interest in receiving health information specific to anxiety. Participants were considered eligible upon meeting the following inclusion criteria: (1) were at least 18 years of age; (2) Lived in the United States or Canada. Two-hundred and fifty subjects were not retained due to providing data in textboxes that were irrelevant to the question posed (e.g. checking ‘other’ in the gender category and providing the name of a city in the corresponding textbox) 14,010 participants were retained for inclusion in data analysis. These participants were on average 55 years old, with 3565 Males, 10,435 Females, and 10 individuals who selected other. Due to the de-identified nature of this study’s data (online survey completion), this study was deemed exempt from oversight by the Brown University Institutional Review Board and informed consent obtention in accordance to federal legislature (US Department of Health and Human Services for the protection of human subjects Sect. 45 CFR 46.104). The experimental procedure is fully described in the Supplementary Materials Section.

### Measures

#### Self-report measures

The self-report questionnaires from Study 1 were used (GAD-7, PSWQ, FFMQ Non-reactivity subscale, MAIA).

#### Self-reported mental health diagnosis

Participants indicated whether they had been diagnosed with either of the following mental health disorders: anxiety, depression, bipolar disorder, schizophrenia/schizoaffective disorder, or other mental health disorder.

#### Demographic variables

See Supplementary Materials Section for a full description of participant demographic information.

### Data analysis

Cluster Analyses procedures are fully described in Supplementary Materials Section.

#### Association between cluster membership and mental health diagnosis

Follow-up chi-square analyses were conducted to determine whether a given cluster had a greater or lower probability of being associated with the label of a mental health diagnosis (a separate model was conducted for each four mental health conditions). A Bonferroni corrected *p* value of 0.0125 was used to account for the 4 different models performed.

#### Between study cluster comparison

To determine similarity between clusters across studies, within-cluster average z-scores for each item were computed and the percent of items between studies that had the same z-score sign (positive/negative z-score, i.e. z-score direction relative to the item’s average across the entire study sample) were obtained.

## Study 1: results

### Cluster analyses determine the presence of 3 psychological phenotypes

**Figure 1:**
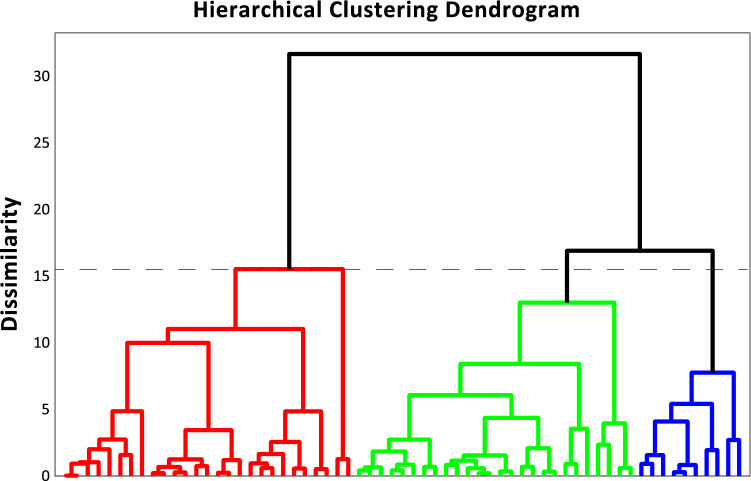
Psychological phenotypic clustering in individuals seeking treatment for anxiety. Dendrogram illustrating the hierarchical agglomerative clustering analysis of questionnaire items from the GAD-7, PSWQ, FFMQ Non-Reactivity Scale, and MAIA questionnaires in Study 1. Different clusters are illustrated in distinct colors (red, green or blue) on the dendrogram (dashed line indicates stopping rule according to the maximal linkage distance).

To determine the presence of clusters in self-report questionnaire data, we used a hierarchical agglomerative approach (Ward’s method, see Supplementary Materials section for complete data analysis procedure details) to determine the number of clusters present. Results from the hierarchical agglomerative clustering approach revealed the presence of 3 clusters (Fig. 1). A *k*-means iterative partitioning approach was then performed to define clusters using *k* = 3 centroids, in order to maximize between-cluster distances and minimize within-cluster distance, yielding silhouette values of 0.503.

To determine items which were significantly distinct between clusters, we conducted one-way ANOVAs with CLUSTER as a between-subjects factor on each questionnaire item’s z-score. Only significantly distinct items are included in cluster description.

**Figure 2:**
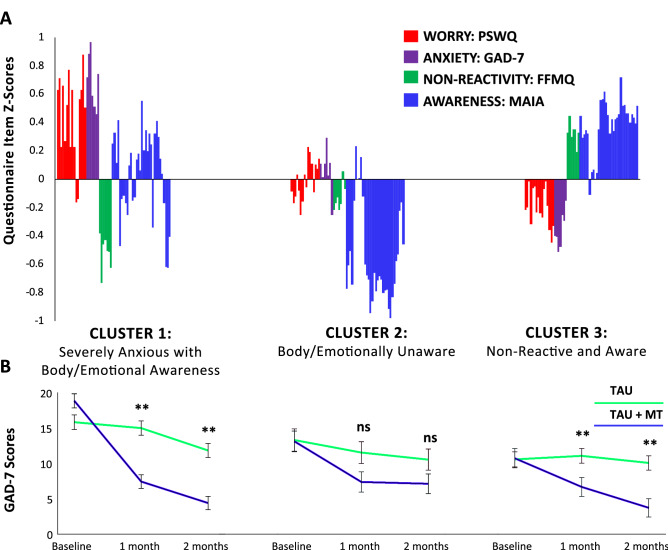
Differential treatment response based on psychological phenotype. (**A**) Study 1 questionnaire item z-scores are shown for Cluster 1 (n = 13), Cluster 2 (n = 21), and Cluster 3 (n = 29): PSWQ: Penn State Worry Questionnaire (red); GAD-7: Generalized Anxiety Disorder 7-item Scale (purple): FFMQ: Non-Reactivity Subscale of the Five Facet Mindfulness Questionnaire (green); MAIA: Multidimensional Assessment of Interoceptive Awareness (blue). (**B**) Study 1 GAD-7 scores are shown at each assessment timepoint (baseline, 1 and 2 months post-treatment initiation) for clusters 1, 2, and 3 (TAU + MT = treatment as usual + mindfulness training; TAU = treatment as usual). Means are shown with standard error, and asterisks denote significant group differences at each assessment timepoint (relative to baseline) as follow-up contrasts from mixed measures ANOVA. **p < 0.001; ns: non-significant.

Figure 2A illustrates questionnaire item z-scores for each cluster. The clusters can be described in terms of intervention (FFMQ, MAIA), mechanism (PSWQ), and outcome (GAD-7) related features (see Table 1).

**Table 1:**
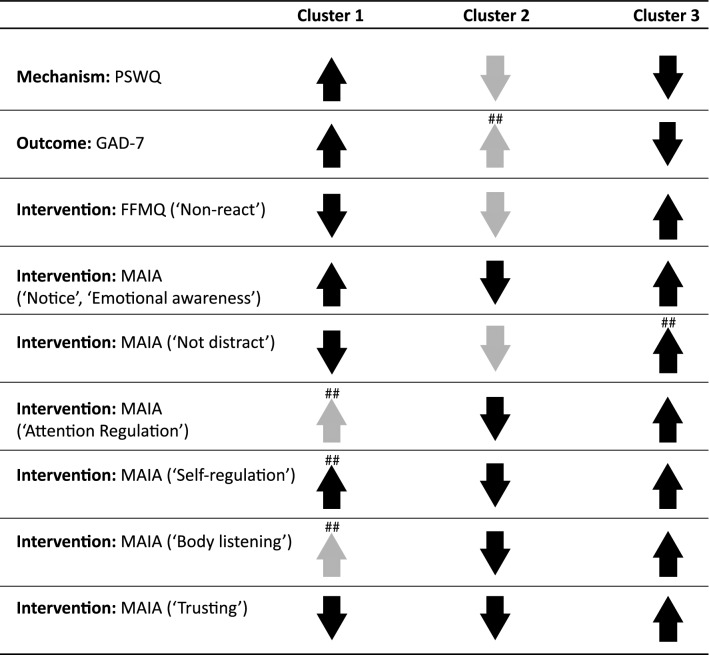
Study 1 cluster description of individual questionnaire items.

Questionnaires’ subscales are listed in parentheses when applicable. Items significantly contributing to cluster formation are included (see results from one-way ANOVA on questionnaire item z-scores with Cluster as a between-group factor, Table S2). 95% confidence intervals for each item’s within-cluster average were computed:

Upward arrows indicate that the within-cluster z-score average for the scale/subscale’s items was positive and that > 50% of items had confidence intervals that exceeded the group mean (full upward arrows) or did not exceed the group mean (light upward arrows).

Downward arrows indicate that the within-cluster z-score average for the scale/subscale’s items was negative and that > 50% of items had confidence intervals that preceded the group mean (full downward arrows) or did not precede the group mean (light downward arrows).

PSWQ: Penn State Worry Questionnaire; GAD-7: Generalized Anxiety Disorder 7-item Scale; FFMQ: Non-Reactivity Subscale of the Five Facet Mindfulness Questionnaire; MAIA: Multidimensional Assessment of Interoceptive Awareness

##Indicate that the direction of > 50% scale/subscale’s items relative to the group mean was distinct between Studies 1 and 2.

Cluster 1 (n = 13) demonstrated the highest scores among all phenotypes on mechanistic and outcome-related variables, and below average scores on mindfulness-related intervention features (FFMQ). Awareness-related intervention features were mixed (MAIA subscales): above-average scores were observed on the majority of features assessing the ability to notice body sensations, emotional awareness, attention regulation, self-regulation, and listening to the body for insight. In contrast, we found below average scores on a subset of items: assessing tendencies to worry about body discomfort and to feel distrustful of or unsafe in their bodies. Bringing these features together, cluster 1 can be summarized as ‘severely anxious with body/emotional awareness’.

Cluster 2 (n = 21) demonstrated scores at or above average on mechanism and outcome-related features, and average scores on mindfulness-related intervention features (FFMQ). This cluster also demonstrated below average scores and lowest of all phenotypes on the majority of awareness-related intervention features (MAIA). Cluster 2 can be summarized as ‘body/emotionally unaware.’

Cluster 3 (n = 29) exhibited the lowest scores of mechanism and outcome-related features, yet the highest scores on intervention-related features of all phenotypes. Cluster 3 can be summarized as ‘aware and non-reactive’.

Table S2 (Supplementary Materials) displays the result of the ANOVAs for each questionnaire item as well as items’ z-scores averages and SDs for each cluster. Cluster composition is more fully described in the Supplementary Materials section.

### Cluster membership is unrelated to demographic variables

To determine any association between cluster membership and demographic variables (age, sex, race, education, income, work and marital status), we conducted a chi-square analysis on categorical variables, and a one-way ANOVAs with CLUSTER as a between-subject factor was conducted on age. No significant association between cluster membership and sex, race, education, income, work nor marital status were found (ps ≥ 0.342). Similarly, no significant difference between cluster membership and age was observed (F(2,60) = 1.86, *p* = 0.165).

### Cluster membership is associated with mental health diagnosis

We found a significant association between cluster membership and the presence of a GAD diagnosis (Pearson chi-square (2) = 12.60, p = 0.002): GAD diagnoses were found in greatest proportion in clusters 1 and 2 (76.9 and 61.9% respectively), followed by cluster 3 (24.1%). No associations were found between cluster membership and other diagnoses, all p’s > 0.13).

### Cluster membership interacts with responses to treatment

To test whether subgroup membership interacted with clinical outcomes, we conducted a TIME (baseline, 1 month, 2 months) X GROUP (TAU + MT, TAU) X CLUSTER mixed measures ANOVA on GAD-7 scores. We found a significant TIME X GROUP X CLUSTER interaction (F(3.75, 106.95) = 2.72, p = 0.037, $\upeta$_p_^2^ = 0.09, observed power = 0.72), indicating that the 2-way interaction of treatment group by time differed depending on which cluster the participants were in, a medium to large effect according to established effect size benchmarks for eta squared^30^.

Follow-up GROUP X TIME ANOVAs within each cluster (alpha for the omnibus F divided by 3 for the three post-hoc ANOVAs performed: 0.05/3 = 0.017) revealed a significant GROUP X TIME interaction for cluster 1 (F(1.77, 19.41) = 31.39, p < 0.001, $\upeta$_p_^2^ = 0.74), and cluster 3 (F(1.77, 47.75) = 13.89, p < 0.001, $\upeta$_p_^2^ = 0.34), but not for cluster 2 (F(1.53, 29.07) = 2.11, p = 0.149, $\upeta$_p_^2^ = 0.10).

Post-hoc within-subjects comparisons (alpha corrected to 0.008 adjusted for 6 contrasts) were performed within each cluster by comparing GAD7 scores between each assessment timepoint (30 and 60 days post-treatment initiation) and baseline for treatment group participants relative to controls. For clusters 1 and 3, these revealed significant contrasts between anxiety scores at the 30 day assessment timepoint and baseline as well as between the 60 day assessment timepoint and baseline for subjects in MT + TAU vs TAU (Cluster1: p < 0.001 for both contrasts; Cluster 3: p = 0.005 comparing GAD7 scores at 30 day vs baseline for MT + TAU vs TAU, p < 0.001 comparing GAD7 scores for 60 day vs baseline for MT + TAU vs TAU). For cluster 2, no significant differences were revealed by the comparisons between anxiety scores at the 30-day assessment timepoint and baseline (p = 0.065) for treatment relative to the control group, as well as the contrast comparing anxiety scores at the 60-day assessment timepoint and baseline (p = 0.215)*.* These data are illustrated in Fig. 2B.

To determine whether group mean values in GAD scores significantly differed between clusters at baseline, *t*-tests (alpha adjusted to 0.017 for 3 between-groups comparisons) comparing GAD scores at baseline between pairs of cluster membership groups were conducted and revealed that cluster1 was higher in baseline anxiety compared to clusters 2 and 3 (*p*s < 0.01), while clusters 2 and 3 were not significantly different from each other when adjusting for multiple comparisons (p = 0.044).

Finally, the TIME (1 month and 2 months post-treatment initiation) X CLUSTER ANOVA on number of mindfulness program modules completed did not reveal any significant main effect of CLUSTER (F(2, 25) = 0.50, p = 0.611, $\upeta$_p_^2^ = 0.04) or TIME X CLUSTER (F(2, 25) = 0.27, p = 0.767, $\upeta$_p_^2^ = 0.02) interaction , indicating no significant differences in engagement measures between clusters across assessment time in the treatment group participants.

Table 2 illustrates means and standard deviations for each cluster by GROUP and assessment time for anxiety and engagement outcome measures.

**Table 2:** Anxiety (GAD-7 scores) and engagement (number of modules completed) by treatment group and cluster at each assessment timepoint.

Outcome	Cluster 1	Cluster 2	Cluster 3
	n = 13	n = 21	n = 29
	M (SE)	M (SE)	M (SE)
Total GAD-7 scores			
TAU + MT			
Baseline	18.86 (1.46)	13.13 (1.37)	10.73 (1.00)
1 Month	7.43 (1.43)**	7.38 (1.34)	6.70 (0.98)**
2 Months	4.40 (1.39)**	7.13 (1.30)	3.72 (0.95)**
TAU			
Baseline	15.83 (1.58)	13.31 (1.07)	10.57 (1.03)
1 Month	15.00 (1.54)	11.54 (1.05)	11.07 (1.01)
2 Months	11.83 (1.50)	10.54 (1.02)	10.07 (0.98)
Number of modules completed			
TAU + MT			
1 Month	14.00 (4.20)	16.38 (3.64)	18.57 (2.75)
2 Months	16.50 (4.70)	20.38 (4.07)	22.36 (3.08)

TAU + MT = treatment as usual + mindfulness training; TAU: treatment as usual; GAD-7: generalized anxiety disorder-7 item scale.

**p < 0.001, follow-up contrasts from mixed measures ANOVA.

## Study 2: results

### Cluster analyses determines the presence of 3 psychological phenotypes

**Figure 3:**
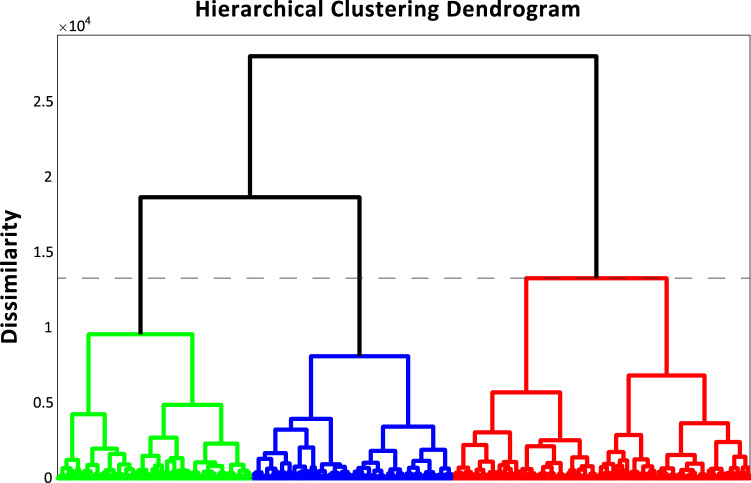
Psychological phenotyping in the general population Dendrogram from the hierarchical agglomerative clustering analysis of questionnaire items from the GAD-7, PSWQ, FFMQ Non-Reactivity Scale, and MAIA questionnaires in Study 2. Distinct colors (red, green or blue) indicate different clusters on the dendrogram (dashed line indicates stopping rule according to the maximal linkage distance).

**Figure 4:**
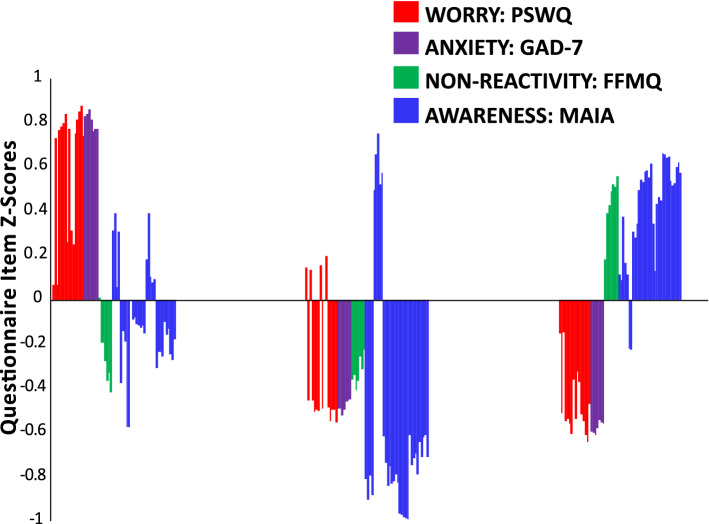
Psychological phenotype composition in the general population. Questionnaire item z-scores for Study 2 participants are shown for Cluster 1 (n = 5629), Cluster 2 (n = 2982), Cluster (n = 5399): PSWQ: Penn State Worry Questionnaire (red); GAD-7: Generalized Anxiety Disorder 7-item Scale (purple): FFMQ: Non-Reactivity Subscale of the Five Facet Mindfulness Questionnaire (green); MAIA: Multidimensional Assessment of Interoceptive Awareness (blue).

As in Study 1, to determine the presence of clusters in self-report questionnaire data, a hierarchical agglomerative approach (Ward’s method) was used, which revealed the presence of 3 clusters (Fig. 3). As performed in Study 1, we then used a *k*-means iterative partitioning approach in order to define clusters using *k* = 3 centroids (mean silhouette values = 0.60, indicative of strong clustering ^31^). Figure 4 illustrates questionnaire item z-scores for each cluster.

Mechanism (PSWQ), outcome (GAD-7), and intervention (FFMQ, MAIA) related features are presented in terms of within-cluster averaged z-scores relative to the entire sample mean (see Table 3 for a summarized description).

**Table 3:**
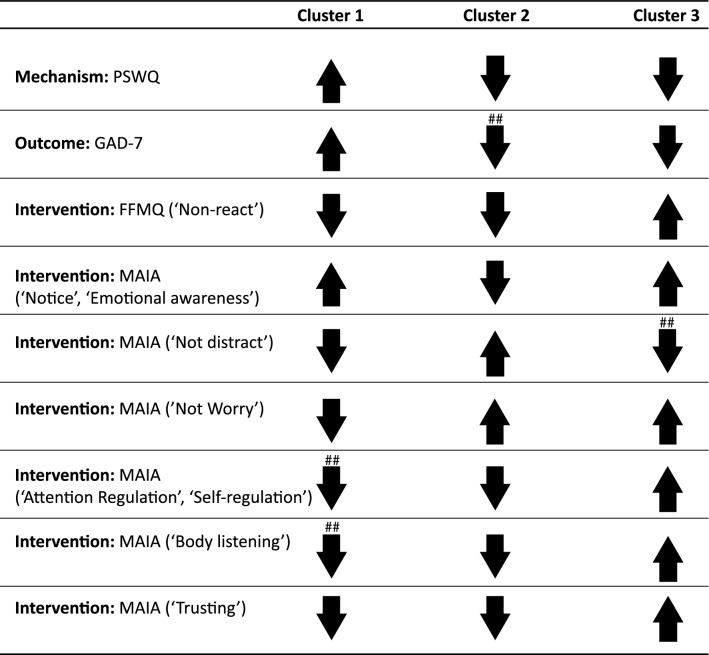
Study 2 cluster description of individual questionnaire items.

Questionnaires’ subscales are listed in parentheses when applicable. 95% confidence intervals for each item’s within-cluster average were computed:

Full upward arrows indicate that the within-cluster z-score average for the scale/subscale’s items was positive and that > 50% of items had confidence intervals that exceeded the group mean. Full downward arrows indicate that the within-cluster z-score average for the scale/subscale’s items was negative and that > 50% of items had confidence intervals that preceded the group mean.

^##^ Indicates that the direction of > 50% scale/subscale’s items relative to the group mean was distinct between Studies 1 and 2.

PSWQ: Penn State Worry Questionnaire; GAD-7: Generalized Anxiety Disorder 7-item Scale; FFMQ_NR: Non-Reactivity Subscale of the Five Facet Mindfulness Questionnaire; MAIA: Multidimensional Assessment of Interoceptive Awareness.

Cluster 1 (n = 5629) scored the highest of all phenotypes in outcome and mechanism-related features. These participants exhibited below average scores on the majority of intervention-related features, and had a mitigated pattern of scores on interoceptive awareness features: above-average scores were observed for MAIA subscales assessing the ability to notice body sensations and emotional awareness. In contrast, below average scores were observed for all other interoceptive awareness subscales (‘not distract’, ‘not worry’, attention regulation, self-regulation, body listening, trust). As in Study 1, this cluster was labeled ‘severely anxious with body/emotional awareness.’ Within-cluster average z-scores indicated a 77.4% similarity with cluster 1 from Study 1 (correlation *r* = 0.78 between the studies’ within-cluster average z-scores).

Cluster 2 (n = 2982) exhibited below-average scores on outcome-related features. They scored below average on the majority of mechanism-related features, with the exception of above-average scores observed for PSWQ features referring to time-related worry, tendency to worry, difficulty in dismissing worrying thoughts, useless worry. These participants also scored below-average on the majority of intervention-related (FFMQ, MAIA) items. As in Study 1, this cluster was labeled ‘body/emotionally unaware’. Within-cluster average z-scores indicated a 72.6% similarity with cluster 2 from Study 1 (correlation *r* = 0.67 between the studies’ within-cluster average z-scores).

Cluster 3 (n = 5399) exhibited the lowest scores of all phenotypes on outcome and mechanism-related features, and the highest on intervention-related features of all phenotypes. As in Study 1, this cluster was labeled ‘aware and non-reactive’.

Cluster composition is described in fuller detail in the Supplementary Materials Section, and Table S3 displays the results of the ANOVAs for each questionnaire item as well as z-scores and SE for each item by cluster. Within-cluster average z-scores indicated a 98.4% similarity with cluster 3 from Study 1 (correlation *r* = 0.92 between the studies’ within-cluster average z-scores).

### Cluster membership is associated with self-reported mental health diagnoses

To determine whether cluster membership was associated with mental health diagnosis (depression, anxiety, bipolar disorder, schizophrenia/schizoaffective disorders), we conducted a chi-square analysis. The proportion of individuals reporting an anxiety diagnosis significantly differed between clusters (Pearson chi-square(2) = 2982.2, p < 0.001), with the largest proportion found in cluster 1 (67%), followed by cluster 2 (21%), and cluster 3 (21%). A similar pattern was found for depression, bipolar disorder, and schizophrenia/schizoaffective disorder diagnoses (Table 4, all p’s < 0.001).

**Table 4:** Study 2 mental health diagnoses and socio-demographic variables for each cluster.

	χ^2a^	Cluster1	Cluster 2	Cluster 3
		n = 5629	n = 2982	n = 5399
		%	%	%
**Mental health diagnoses**				
Anxiety	2982.17**	67.00	21.00	20.90
Depression	2673.04**	67.60	27.80	21.90
Bipolar	1140.46**	27.90	7.90	6.50
Schizophrenia/schizoaffective	262.57**	7.20	1.90	1.60
**Sex**	365.57**			
Male		16.90	32.80	30.20
Female		83.00	67.10	69.70
Other		0.100	0.00	0.10
**Living area**	98.01**			
Urban		25.50	27.10	28.30
Suburban		28.50	34.70	34.10
Rural		46.00	38.20	37.60
**Education**	345.12**			
Middle School		5.10	3.70	2.30
High School		52.50	50.70	41.70
College/Associate degree		36.20	35.00	41.80
Bachelor		4.00	6.70	8.70
Postgraduate		2.10	3.90	5.50
**Income**	271.17**			
< 25,000		65.00	55.70	50.60
25,000–49,999		20.60	24.20	25.90
50,000–74,999		5.40	7.90	9.70
75,000–99,999		1.80	2.90	3.50
100,000–199,999		0.80	1.50	1.70
≥ 200,000		0.20	0.40	0.30
No answer		6.10	7.50	8.20
Age**		50.86 (0.16)	58.13 (0.21)	57.53 (0.16)

Except for Age which includes M(SE), within-cluster % of participants are reported.

**p < 0.001.

Significant between-cluster age difference, F(2,14,007) = 585.34, *p* < 0.001.

^a^Pearson Chi-square statistics are reported.

### Cluster membership is associated with demographic variables

To determine whether cluster membership was associated with demographic variables (age, sex, income, education, living area), we conducted chi-square analyses with categorical variables and one-way ANOVAs with the continuous variable of age. These revealed a significant association between cluster membership and sex (Pearson chi-square(2) = 365.6, p < 0.001), with the largest proportion of females within cluster 1 (83%), followed by cluster 3 (70%) and 2 (67%).

We found a similar pattern of results for other demographic variables (see Table 4), such that participants in cluster 1 were more likely to report living in rural areas (46% vs 38% in both clusters 2 and 3 respectively), have lower income (65% reporting earning < $25,000 yearly, vs 56% and 51% in clusters 2 and 3 respectively), and were overall younger (M = 51 years ± 11.1) than participants in clusters 2 and 3 (all ps < 0.001) who were both on average 58 years of age (± 12). A significant association between cluster membership and education was also found (see Table 4) such that the proportion of participants with lower education was greatest in cluster 1, followed by clusters 2, and 3 (5% in cluster 1 reporting having middle school as their highest education level, vs 4% and 2% in clusters 2 and 3 respectively).

### Feature reduction

To determine a reduced number of features preserving the composition of clusters, we selected features with maximal amount of variance between clusters in Study 2’s dataset (due to its larger sample size) that had the largest effect sizes from the one-way ANOVAs using cluster as a between-group factor on questionnaire data items’ z-scores. We conducted a cluster analysis on the smaller set of features by applying a PCA to reduce the data to 2 dimensions, followed by a hierarchical agglomerative clustering and k-means analysis (the same set of analysis steps as in Studies1 and 2). This was conducted on half, one third, and one quarter of items within each scale. The solution with the lowest number of features that preserved cluster composition for the majority of features and had the highest mean silhouette value (0.570) was that for one-third of items from each scale (total of 19 items), which yielded comparable mean silhouette values (0.573) when applied to data from Study1 (using this reduced set of 19 features identified in Study 2 from between-cluster effect sizes). These items are highlighted in Table S3.

## Discussion/conclusion

This study demonstrated that low-burden, minimal-cost, self-report methods can accurately identify psychological phenotypes based on characteristics related to treatment (mindfulness, interoceptive awareness), mechanism (worry) and outcomes (anxiety) in individuals seeking treatment for anxiety (Study 1), and general populations (Study 2). We found that these phenotypes also constitute a marker of mental illness susceptibility (Studies 1 and 2) and treatment response (Study 1). These results demonstrate the proof-of-concept that rapid, low-cost methods can be employed to match individuals with treatment to optimize outcomes—for example, mindfulness training for anxiety can be tailored to particular subgroups’ baseline psychological phenotype.

Our results show high degree of similarity in cluster-based solutions (between cluster correlations of 0.67–0.92 across studies) in both treatment-seeking individuals and at the general population level for psychological attributes with characteristics relevant to a disorder’s symptomatology, mechanistic factors, and type of psychological treatment. In addition, cluster membership significantly interacted (moderate to large effect size) with anxiety symptom improvement from our intervention (Study 1). In essence, clinicians could benefit from this information by recommending this type of mindfulness-based intervention as higher-line treatment options to anxious patients with interoceptive awareness (Cluster 1) or lower anxiety/worry with high awareness (Cluster 3) as they showed large effect sizes of symptom improvement, but opt for a different treatment selection for participants with some anxiety/lower interoceptive awareness (Cluster 2). Future studies optimizing this Cluster 2’s outcome could further guide recommendations for participants with this type of baseline psychological profile.

In Study 1, although baseline differences in anxiety were observed between clusters (with highest anxiety scores observed in Cluster 1, followed by Cluster 2, and with Cluster 3 having lowest anxiety scores), Cluster 2’s non-significant outcome improvement from the intervention was unlikely due to baseline GAD score differences. As such, although anxious patients with interoceptive awareness (Cluster1) was higher in baseline anxiety compared to Clusters 2 and 3, clusters 2 and 3 were not significantly different from each other in baseline GAD scores. The cluster that did not show significant responses to the intervention was Cluster 2, which indicates that the latter effect was not explained by differences in baseline GAD levels between clusters because the cluster with lowest baseline anxiety (Cluster 3) showed significant improvement from the intervention.

The results of these studies could also guide future research in a number of ways: first by applying the same clustering analysis framework to implement personalized medicine approaches to other mental health disorders by using attributes relevant to the disorder’s symptomatology, mechanistic factors, and characteristics of the intervention. Brain and physiological correlates of psychological phenotypes could be studied as well. Together, this type of framework could enhance the application of personalized medicine into clinical mental health settings, where heterogeneity to treatment is present and empirically-based guidelines are lacking for treatment selection based on individual characteristics^6^. The low-cost aspect, accessibility, and speed of acquisition of combining validated psychological assessment tools could contribute to the feasibility in implementing such phenotyping to clinical mental health settings to facilitate personalized treatment selection or preventive measures recommendations. Implications are discussed with respect to these study’s limitations and future study directions as well as potential personalized medicine applications.

### Impact on psychological phenotyping and personalized medicine

The results of this study indicate that the use of psychological variables related to an intervention’s modality, mechanism(s) of action and outcome(s), can be used to characterize individuals into subgroups that are associated with mental health diagnoses and treatment outcomes. We demonstrated that incorporating key psychological markers of intervention and mechanism of change can identify cluster membership that has clinically-relevant impacts on outcomes for individuals with anxiety. The same approach can be applied to personalize treatment in other mental health conditions in which theoretical aspects of mechanism and treatment have already been identified (e.g. cognitive-behavioral therapy, depression and changes in cognitive patterns^32^).

Our results demonstrate a potentially important advance for personalized medicine: development of individually-targeted treatment can be enhanced to optimize intervention effectiveness, and/or individualize treatment selection. For example, individuals in the ‘body/emotionally unaware’ cluster (cluster 2 in Study 1) that showed non-significant clinical outcome improvement relative to clusters 1 and 3 may need the incorporation of treatment components targeting interoceptive awareness skills (e.g. yoga) and/or may benefit from trying a different treatment modality (e.g. CBT). Additionally, it will be important to establish this subgroup’s response to medication treatment and/or whether these individuals are more generally psychologically treatment resistant, necessitating stepped-up care immediately upon the beginning of treatment (e.g. combining individual psychotherapy with a digital therapeutic).

The results of these studies also contribute to simple, low-cost methods for the identification of individuals who may be at-risk for mental illness, thereby promoting the development of more effective prevention measures. For example, the higher anxiety clusters identified in both studies exhibited a greater proportion of participants with mental health disorders, and may particularly benefit from engaging in prevention measures (e.g. physical exercise, healthy eating habits^33,34^).

### Practical utility in clinical settings

Studies in the pursuit of personalized medicine have largely focused on biomarker identification methods which are costly, time- and labor-intensive. For example, cluster analysis of functional magnetic resonance imaging datasets has contributed to the identification of brain-based, mechanistically derived subgroups in clinical populations—e.g. clusters exhibiting distinct functional connectivity in particular brain networks in people with schizophrenia^35^. While the establishment of objective biomarkers is essential to the application of personalized medicine, these require infrastructure and technological expertise that are largely high-cost and limited in access. Nonetheless, reports on psychological phenotyping involving the use of machine learning and psychometric data show promise^14,36,37^. Our findings not only set the stage to guide and synergize with biomarker studies (e.g. combine psychological phenotyping with brain region clustering in functional neuroimaging studies), but also have the advantage of practical utility in clinical settings. Psychological phenotyping can be developed and deployed rapidly, and at low cost: someone with anxiety can fill out questionnaire items—in less than ten minutes—at home or on their smartphone in a clinic waiting room. The data can then be algorithmically cluster-analyzed and delivered to their electronic medical record in real-time, where it can be viewed by a clinician to scientifically guide and inform treatment selection in a personalized manner.

### Limitations and future directions

The present studies are not without limitations. Despite the observed large effect sizes for the interaction of cluster membership with treatment response in Study 1^30^, these results should be interpreted with caution and warrant replication in future larger studies. With respect to analyses between cluster membership and associations with mental health diagnoses, the limited sample size from Study 1 may have masked the presence of significant effects, while conversely, the large sample size from Study 2 may have yielded significant differences with less clinical relevance. Moreover, despite convergence of results between studies in the association between cluster membership and mental health diagnosis, the differences in diagnosis assessment (diagnosed by an experimenter in Study 1, and self-reported in the larger Study 2 sample) also limits interpretations. Future replication of these analyses is therefore warranted to address these limitations.

In addition, although clusters showed a large confluence of features between both studies (73–98% and between cluster correlations of 0.7–0.9), some distinctions were observed, particularly for the first phenotype on intervention (awareness) related features, and for the second phenotype on outcome/mechanism–related features. It is possible that these differences stem from population differences in baseline anxiety: Study 1 was a treatment-seeking sample with on average moderate levels of anxiety, whereas Study 2 was a larger sample taken from the general population with on average mild levels of anxiety. Moreover, the use of adaptive intervention designs^5^ manipulating specific aspects of interventions (e.g. duration, type, dose) may help to identify key features (e.g. experiential avoidance in the case of anxiety^38^ and inform intervention development to bolster treatment outcomes in subgroups that may otherwise not be predicted to respond as robustly. Finally, the use of the reduced data set features yielding comparable strength of clustering and cluster composition across studies would facilitate computation costs when analyzing large datasets as well as speed in data collection with preserved clustering accuracy, yet this result warrants replication in future independent datasets.

In conclusion, the results of these studies supported the clinical practical utility of psychological phenotyping using machine learning and self-report questionnaire data involving characteristics related to a psychological treatment’s modality, mechanism of action, and outcome. In other words, machine learning can be used to identify psychological phenotypes, or subgroups of people based on baseline psychological characteristics relevant to a particular disorder that can inform patients’ response to its treatment or psychological disorder susceptibility and have implications in terms of the use of personalized medicine in clinical mental health practice*.* The application of this framework also has high practical utility in such settings (ease, rapidity, accessibility and low cost). Finally, these results set the stage for future studies incorporating neurobiological data to determine the correspondence between psychological phenotypes and underlying neurobiological correlates/markers.

## Supplementary Information

Supplementary Information.

## Data Availability

Data included in the present article will not be made publicly available due to resource sharing specifications included in the NIMH/NIH grant supporting this project. Brown University will adhere to the NIH Grants Policy on Sharing of Unique Research Resources including the “Sharing of Biomedical Research Resources: Principles and Guidelines for Recipients of NIH Grants and Contracts” issued in December, 1999 and the Data Sharing Policy (section 8.2.3.1) from the October 2019 revision. Specifically, material transfers would be made with no more restrictive terms than in the Simple Letter Agreement or the UBMTA and without reach through requirements. Nonetheless, data access requests from academic investigators can be directed to the corresponding author.
