# Supplementary material for: Cluster-based psychological phenotyping and differences in anxiety treatment outcomes
Source: Sci Rep. 2023 Feb 21;13:3055. doi: 10.1038/s41598-023-28660-7 (PMC9944281; doi:10.1038/s41598-023-28660-7)
Supplement: Supplementary file 1 — Supplementary Information. [file 41598_2023_28660_MOESM1_ESM.docx]

Supplementary Materials

**Study 1: Methods and Materials**

*Participants*

Among the 167 assessed for eligibility, 65 eligible participants were elected to participate and enrolled in the study. Upon providing informed consent, participants were randomly assigned to one of two arms: continue treatment as usual and complete the app-based MT program (TAU+MT), or to continue treatment as usual (TAU). Among those who were enrolled in the study and underwent random group allocation, two withdrew their participation due to personal reasons, leaving a total of 63 participants having completed baseline assessments, of which 61 participants completed all follow-up assessments at 1 and 2 months post-intervention initiation. A Consolidated Standards of Reporting Trials (CONSORT) diagram illustrating the participant flow process is shown in Figure S1, and participant characteristics are displayed in Table S1.

*Inclusion Criteria for Participant Recruitment*

Screening interviews were conducted by telephone using the following inclusion criteria: 1) owning a smartphone 2) having a GAD-7 score of 10 or above (a suggested benchmark score for a GAD diagnosis) [1]; 3) indicated consent for the reception of check-in calls; and (4) were at least 18 years of age. In addition, participants were excluded upon meeting any of the following criteria: 1) having altered their dosage on a psychoactive medication in the past two months; 2) use of benzodiazepines and/or hypnotic drugs for sleep-related purposes; 3) having a history of a psychotic disorder or of bipolar disorder, (4) having any severe medical condition with the potential to interfere with the completion of the study, 5) living with another participant concurrently enrolled in the study 6) having previously used other similar/related apps.

*Intervention*

The app-delivered MT program (Unwinding Anxiety), as used in the article published by Roy and colleagues (2021) [2] was designed to help people manage anxiety by teaching skills to develop awareness of maladaptive thought-feeling cycles that perpetuate anxious worry [2]. In addition, skills are taught to bring mindful attention to states of stress and worry as they occur in the present-moment, fostering the uncoupling of anxious states and reactive worry thought patterns. The program consists of over 30 daily modules, which include short didactic and mindfulness training materials: videos/ animations (approximately 10 minutes/day), guided meditations initiated by the user (5-15 minutes), regular check-ins, and short mindfulness exercises (~30 seconds) initiated by the user to help disrupt anxiety patterns as they occur in the present-moment. Themes and content of the program are described in a previous report [2]. This program was developed using a combination of clinical experience related to anxiety treatment and expertise in the development of other similar app-based MT programs that have yielded significant reductions in health-detrimental behavior (smoking, maladaptive eating) [3-5]. The use of this MT program was recently shown to have clinically meaningful outcomes: a 57% GAD-7 score reduction was observed among anxious physicians in a pilot study after using the program for three months [6].

*Measures*

*Generalized Anxiety Disorder 7-item (GAD-7).* This scale is a 7-item self-report questionnaire used in clinical settings to determine probable diagnosis of GAD and monitor the severity of the disorder’s symptoms. The scores on this scale (ranging from 0 to 21) are indicative of different levels of symptom severity: 5 = mild, 10 = moderate, and 15 = severe anxiety symptoms [7]. The GAD-7 has an internal consistency score of Cronbach α = .92 [7], and scores above 10 were shown to have sensitivity and specificity of 89 and 82% respectively in the detection of a GAD diagnosis [1, 7]. This tool is highly correlated with the Hamilton Anxiety Scale (HAM-A, *r*=0.852) and strongly predictive of disability in primary care patients [1] along with a high likelihood of accurately detecting likely GAD diagnoses in patients [8].

*Penn State Worry Questionnaire (PSWQ).* Worry has been previously identified as a key component of anxiety [9] . It has also been shown to mechanistically ‘drive’ anxiety through negative reinforcement (e.g. as a means to avoid negative affective states, which could be perceived as threatening) [10]. The 16-item PSWQ questionnaire is a tool used to assess worry; it has high internal consistency (Cronbach α = .93) and good test-retest reliability [11].

#### Non-Reactivity Subscale of the Five Facet Mindfulness Questionnaire (FFMQ). The FFMQ is commonly used to measure self-reported mindfulness. The non-reactivity subscale has previously been shown to mediate the effects of MT on anxiety outcomes [2]. This subscale includes 7 items from the FFMQ, a 39-item questionnaire assessing different dimensions of trait mindfulness. Specifically, the non-reactivity subscale assesses the extent to which individuals are reactive to their experiences (e.g. thoughts, images, feelings) [12]. Its internal consistency is of Cronbach α = .75 [13].

#### Multidimensional Assessment of Interoceptive Awareness (MAIA). This questionnaire (Cronbach α=.66-.87) comprises 32 questions assessing interoceptive awareness –a key component of MT. More particularly, the following 8 facets of interoceptive awareness are assessed in separate subscales: noticing (awareness of body sensations), ‘not’ - distract (tendency to refrain from distraction to manage uncomfortable body sensations), ‘not’- worry (tendency to not be worried or distressed about uncomfortable body sensations), attention regulation (extent to which subjects can regulate, direct and sustain attention to sensations), emotional awareness (extent to which subjects can identify physiological emotional processes with bodily sensations), self-regulation (ability to manage distressing states by directing attention towards sensations of the body), body-listening (extent to which one listens to the body to obtain insight), trusting (extent to which one experiences a sense of safety and trust in the body) [14].

*Procedure*

After undergoing telephone screening interviews for study eligibility, participants were invited to the laboratory where they provided written informed consent prior to enrolling in the study. Next, they were assessed using the MINI International Neuropsychiatric Interview (abbreviated version) by the project director, previously trained and receiving supervision from a psychiatrist. This initial assessment was to confirm the probable diagnosis of GAD and evaluate the presence of other potential comorbid psychiatric conditions (major depressive disorder, specific phobias, post-traumatic stress disorder, obsessive-compulsive disorder, social anxiety disorder, agoraphobia, panic disorder).

Participants were then asked to complete online surveys using Qualtrics, which included assessments of demographic information as well as self-report questionnaires (see Measures below).

Following this baseline survey completion, participants were randomly assigned to their allocated group. To do this, each participant was given a sealed envelope containing group allocation information, which had been prepared and sent by individuals who were independent of the study team. An independent statistician generated the randomization procedure, a 1:1 randomization scheme with variable block sizes of 4 and 6. Members of the team who were involved in the group allocation process and performing assessments did not conduct any of the statistical analyses. The principal investigator and the statistician who conducted the statistical analysis were blinded to group until all analyses were complete.

For participants assigned to TAU+MT, assistance was provided in downloading the app and becoming familiar with the features. They were given instructions of completing 1 app module per day for the following 30 calendar days at the time they determined. The app was set to deliver check-ins (which could be changed if desired) and offering of brief mindfulness exercises. Other app feature use was encouraged but stated as not mandatary for study participation. Check-in text messages were sent on the 3^rd^, 7^th^, 14^th^, and 21^st^ days of the study by the project director to encourage motivation and engagement, and offer assistance with any potential technical difficulties.

After initiation of the intervention, participants were prompted via email to complete online self-report questionnaires at 1 and 2 months. A $10 Amazon gift card was given as remuneration for completion of the baseline survey and $35 for each additional survey completed (1 and 2 months, up to $80).

*Data Analysis*

*Cluster Analyses.* A principal component analysis (PCA) was applied on the total of 62 features (total items from the PSWQ, GAD7, FFMQ, MAIA) to reduce the dimensionality of the data as a pre-processing step prior to conducting cluster analyses [15, 16], using Matlab computing software [17], onto standardized z-scores due to the different numerical scales used between questionnaires. Next, a hierarchical agglomerative cluster analysis onto the first two retained principal components (as determined by the eigen value scree plot) was conducted using Matlab [17], Ward’s method [18], and the Euclidean distance as the distance metric. The maximal distance value between hierarchy levels was set as the cutoff criterion or stopping rule to determine the number of clusters, i.e. the number of clusters retained were those having been formed at the hierarchical stage below which the maximal distance difference between links was observed [19, 20].

Finally, k-means clustering [21] was conducted using a predefined number of centroids determined from the agglomerative clustering approach.

*Interaction between subgroup membership and responses to MT.* The main effect of GROUP on outcome variables is the focus of another report [2] and is not included here, because the goal of the present research was to determine the moderating role of cluster membership on treatment outcomes. A Greenhouse-Geisser correction criterion was applied to account for sphericity of repeated measures. Unless otherwise specified, significance was determined using a *p* < 0.05 threshold. Partial eta squared measures of effect sizes are included for which the following cutoff criteria have been established (0.01 = small, 0.06 = medium, 0.14 = large) [22].

**Study 2: Methods and Materials**

*Procedure*

Potential participants were recruited by Sharecare Inc., via email inviting individuals to participate in research. Participants were screened for eligibility via an online survey in Qualtrics. Those who were deemed eligible were directed to another page to enter demographic information and fill out a short battery of self-report questionnaires.

*Measures*

*Demographic Variables.* The following demographic variables were assessed: age, sex (male, female, other), living area (by answering ‘Urban’, or ‘Suburban’, or ‘Rural’ to the question “Which of the following best describes the area in which you reside?), education (by answering ‘Middle School’, ‘High School’, ‘Some College/Associate’s Degree’, ‘Bachelor’s Degree’, or ’Post-graduate degree’ to the question ‘What is your highest education level completed?’), and income (by answering ‘<$25K’, ‘ $25K-49,999‘, ‘$50K-74,999’, ‘$75K-99,999’, ‘$100K-$199,999’, ‘$200K’, ‘Prefer not to answer’ to the question ‘What is your household annual income?’).

*Data Analysis*

*Cluster Analysis.* The same data analysis steps as those used in Study1 were applied to the Study 2 sample. First, we applied a PCA onto the total of 62 item features from the four questionnaires (standardized into z-scores) to reduce the number of dimensions in the data prior to performing cluster analyses [15, 16], following which a hierarchical agglomerative clustering procedure (using Ward’s method and the Euclidean distance) was applied onto the first two principal components (as determined from the eigen value scree plot). As in Study1, the number of clusters were determined using the maximum between-cluster distance as the stopping rule, and k-means cluster analysis was then applied onto the reduced dataset using the determined number of clusters. To determine whether GAD7 and PSWQ had problematic collinearity levels, we used logistic regression in both studies to examine multicollinearity between both variables in predicting an anxiety disorder diagnosis. Both PSWQ and GAD7 did not have a variance inflation factor that was substantially greater than 1 (<3 for both studies), therefore not indicating problematic multi-collinearity between predictors [23]. While the two variables of PSWQ and GAD were related, there remained a wide portion of variance in one variable not accounted for by the other (79% and 42% of the variance in GAD-7 was unique from that of PSWQ scores for Studies1 and 2 respectively, determined by regressing PSWQ scores onto GAD-7 scores in each study). Thus, removing either of these from the features included in the clustering analyses may have led us to miss features with unique contributions to cluster formation relevant to treatment responses.

**Study1: Results**

*Cluster Description*

The clusters can be described as follows for questionnaire items significantly contributing to cluster formation (see Table S2). Cluster 1 (n =13) demonstrated highest scores among all phenotypes on worry (PSWQ feature z-score range: 0.5-0.9 SD above the mean), and anxiety feature (GAD-7 feature z-scores range: 0.6-1 SD above the mean). It also had the lowest scores among all phenotypes on non-reactivity to experience (FFMQ z-score range: 0.4 to 0.7 SD below the mean). Cluster 1’s patterns of scores on awareness were mitigated depending on the MAIA subscale: their scores were near or above average on body awareness (0.1-0.4 SD above the mean for ‘noticing’ subscale items), their ability to regulate attention (0.06-0.6 SD above the mean and 0.1-0.2 SD below the mean for ‘attention regulation’ subscale), attribute sensations to manifestations of emotions (0.2-0.3 SD above the mean for ‘emotional awareness subscale’). They also scored near or above average on the majority of features for the ability to self-regulate (0.3-0.4 SD above the mean except for one item at 0.3 below the mean in the ‘self-regulation’ subscale). Finally, Cluster 1 exhibited tendencies to be distracted by body discomfort (0.5 SD below the mean), to not feel a sense of trust or safety in the body (0.4-0.6 SD below the mean) or listen to their body for insight (0.2 SD below the mean and 0.04 – 0.2 SD above the mean for the ‘body listening’ subscale), as indicated by their below or near average scores on the ’not distract’, ‘trusting’ and ‘body listening’ subscale items. Cluster 1 can be summarized as ‘severely anxious with body/emotional awareness’.

Cluster 2 (n = 21) had a varied pattern of scores on different worry items. Their scores were near or below average for items related to overwhelming/constant worry, worrying about completing tasks and to finish them in time, generalized worry (across many situations) (items 1-2,4,7,9 on the PSWQ, z-score range: 0.03 above the mean – 0.2 SD below the mean). Their scores were above or near average on items specific to unstoppable worry, worry as a general trait, and worrying about projects until their completion (PSWQ items 14-16 z-score range 0.07- 0.1 above the mean). With respect to anxiety, Cluster 2’s scores were near or above average on most GAD-7 items (z-score range for items 1-4: 0.02-0.3 above the mean) and were below average on the last GAD-7 item related to the tendency to ‘feel afraid as though something awful might happen’ (0.3 SD below the mean for item 7). With respect to non-reactivity to experience, they scored below or near average on FFMQ items (0.07-0.2 SD below the mean). Finally, Cluster 2 had the lowest scores of all phenotypes on interoceptive awareness items, with scores below the mean for MAIA items (z-score range: 1 - 0.2 below the mean). Cluster 2 can be summarized as ‘body/emotionally unaware.’

Finally, Cluster 3 (n = 29) had the lowest scores of all phenotypes on worry (PSWQ items z-score range: 0.2 to 0.5 below the mean) and anxiety (GAD-7 z-score range 0.2 – 0.5 below the mean). In contrast, this cluster exhibited the highest scores of all phenotypes on non-reactivity (FFMQ items z-score range: 0.3 – 0.5 above the mean) and interoceptive awareness (MAIA items z-score range: 0.3-0.7 above the mean). Cluster 3 can be summarized as ‘aware and non-reactive’. Table S2 displays ANOVA results, as well as within-cluster average z-scores and SE for each feature.

**Study2: Results**

*Cluster Description*

Cluster 1 (n = 5,629) scored the highest of all phenotypes on worry (PSWQ z-score range: 0.07-0.9 SD above the mean) and anxiety (GAD-7 z-score range: 0.8-0.9 SD above mean). Cluster 1 scored below or near average for non-reactivity items (FFMQ z-score 0.4 SD below the mean to 0.01 SD above the mean). With respect to interoceptive awareness, scores on awareness were below or above average depending on the MAIA subscale. Cluster 1 scored above-average on body awareness (‘noticing’ subscale z-score range: 0.06 – 0.4 SD above the mean) and the ability to attribute bodily sensations to manifestations of emotions (emotional awareness subscale, z-score range: 0.1 – 0.4 SD above the mean). In contrast, Cluster 1 exhibited scores below average for the ‘not distract’ (z-score range: 0.1 – 0.4 SD below the mean) and attention regulation subscales (z-score range: 0.1 – 0.2 SD below the mean). These participants also exhibited below average scores on self-regulation (z-score range: 0.2-0.3 SD below the mean), the ‘body-listening’ subscale (z-score range: 0.1 – 0.2 SD below the mean), and having a feeling of safety / trust in body (z-score range: 0.2 – 0.3 SD below the mean). Finally, Cluster 1 exhibited scores below average on the ‘not worry’ subscale items (z-scores = 0.01-0.6 SD below the mean). As in Study1, Cluster 1 can be summarized as ‘severely anxious with body/emotional awareness’.

Cluster 2 (n = 2982) exhibited scores above average on certain worry items (PSWQ items 1,3,8,11) pertaining to time-related worry, tendency to worry, difficulty in dismissing worrying thoughts, useless worry (z-score range: 0.1 – 0-.2 SD above the mean). Cluster 2 also exhibited scores on the other PSWQ items that were average or below average (z-score range: 0 - 0.6 SD below the mean), as well as anxiety (GAD-7 items’ z-score range: 0.5-0.5 SD below the mean). Cluster 2’s scores were below average on non-reactivity items (FFMQ items z-score range: 0.2-0.4 SD below the mean). Finally, Cluster 2 exhibited different patterns of awareness scores depending on the MAIA subscales. Their scores were above average for items pertaining to the ability to refrain from distraction to manage bodily discomfort (not distract subscale, z-score range: 0.5-0.8 SD above the mean) and to not worry about uncomfortable body sensations (MAIA items 8,9 from the ‘not worry’ subscale z-scores: 0.5-0.6 SD above the mean). In contrast, Cluster 2 displayed scores below average for all other interoceptive awareness items (z-score range: 0.6-1 SD below the mean). As in Study1, Cluster 2 can be summarized as ‘body/emotionally unaware.’

Cluster 3 (n = 5,399) scored the lowest of all phenotypes on worry (PSWQ items’ z-score range: 0.15-0.6 SD below the mean) and anxiety (GAD-7 items’ z-score range: 0.5-0.6 SD below the mean), and the highest on non-reactivity (0.2-0.6 above mean). With respect to interoceptive awareness, except for 2 items of the ‘non-distract’ scale (z-score range: 0.2 SD below the mean) pertaining to the use of distraction to manage uncomfortable sensations, Cluster 3 exhibited the highest scores of all phenotypes on the majority of interoceptive awareness items (z-score range: 0.1- 0.7 SD above the mean). Table S3 displays ANOVA results, as well as within-cluster average z-scores and SE for each feature. As in Study1, Cluster 3 can be summarized as ‘aware and non-reactive’.

References

1. Kroenke, K., et al., *Anxiety disorders in primary care: prevalence, impairment, comorbidity, and detection.* Ann Intern Med, 2007. **146**(5): p. 317-25.

2. Roy, A., et al., *Clinical efficacy and psychological mechanisms of an app-based digital therapeutic for generalized anxiety disorder.* J Med Internet Res, 2021. **23**(12): p. e26987.

3. Janes, A.C., et al., *Quitting starts in the brain: a randomized controlled trial of app-based mindfulness shows decreases in neural responses to smoking cues that predict reductions in smoking.* Neuropsychopharmacology, 2019. **44**(9): p. 1631-1638. PubMed PMID: PMC6785102.

4. Brewer, J.A., et al., *Mindfulness training for smoking cessation: results from a randomized controlled trial.* Drug Alcohol Depend, 2011. **119**(1-2): p. 72-80. PubMed PMID: PMC3191261.

5. Mason, A.E., et al., *Testing a mobile mindful eating intervention targeting craving-related eating: feasibility and proof of concept.* J Behav Med, 2018. **41**(2): p. 160-173. PubMed PMID: PMC5844778.

6. Roy, A., et al., *Physician Anxiety and Burnout: Symptom Correlates and a Prospective Pilot Study of App-Delivered Mindfulness Training.* JMIR Mhealth Uhealth, 2020. **8**(4): p. e15608. PubMed PMID: PMC7160707.

7. Spitzer, R.L., et al., *A brief measure for assessing generalized anxiety disorder: the GAD-7.* Arch Intern Med, 2006. **166**(10): p. 1092-7.

8. Swinson, R.P., *The GAD-7 scale was accurate for diagnosing generalised anxiety disorder.* Evid Based Med, 2006. **11**(6): p. 184.

9. Association, A.P., *Diagnostic and statistical manual of mental disorders (DSM-5)*. 5th Edition ed. 2013, Arlington, VA: American Psychological Association.

10. Borkovec, T.D., W.J. Ray, and J. Stöber, *Worry: A Cognitive Phenomenon Intimately Linked to Affective, Physiological, and Interpersonal Behavioral Processes.* Cognitive Therapy and Research, 1998. **22**(6): p. 561-576.

11. Meyer, T.J., et al., *Development and validation of the Penn State Worry Questionnaire.* Behav Res Ther, 1990. **28**(6): p. 487-95.

12. Baer, R.A., et al., *Using self-report assessment methods to explore facets of mindfulness.* Assessment, 2006. **13**(1): p. 27-45.

13. Baer, R.A., et al., *Construct validity of the five facet mindfulness questionnaire in meditating and nonmeditating samples.* Assessment, 2008. **15**(3): p. 329-42.

14. Mehling, W.E., et al., *The Multidimensional Assessment of Interoceptive Awareness, Version 2 (MAIA-2).* PLoS One, 2018. **13**(12): p. e0208034. PubMed PMID: PMC6279042.

15. Embrechts, M., et al., *Hierarchical Clustering for Large Data Sets*, in *Advances in Intelligent Signal Processing and Data Mining: Theory and Applications* P. Georgieva, L. Mihaylova, and C.J. Lakhmi, Editors. 2012, Springer: NY, USA.

16. Ding, C., et al., *Adaptive dimension reduction for clustering high dimensional data*, in *Second IEEE International Conference on Data Mining. Proceedings.* 2002. p. 147-154

17. Matlab, *9.6.0.1150989 (R2019a)*. 2019, The MathWorks Inc.: Natick, Massachusetts.

18. Ward, J.H., Jr., *Hierarchical Grouping to Optimize an Objective Function.* Journal of the American Statistical Association, 1963. **58**: p. 236-244.

19. Milligan, G.W. and M.C. Cooper, *An examination of procedures for determining the*

*number of clusters in a data set.* Psychometrrika, 1985. **50**: p. 159-179.

20. Clatworthy, J., et al., *The use and reporting of cluster analysis in health psychology: a review.* Br J Health Psychol, 2005. **10**(Pt 3): p. 329-58.

21. Lloyd, S.P., *Least squares quantization in PCM.* IEEE Transactions on Information Theory, 1982. **28**(2): p. 129-137.

22. Cohen, J., *Statistical power analysis for the behavioral sciences (2nd ed.)*. 1988, Hillsdale, NJ: Lawrence Erlbaum Associates

23. Bowerman & O’Connell, 1990, Linear Statistical Models: An applied Approach (2^nd^ Ed), Belmont, Ca: Duxbury

*Table S1. Participant Characteristics by Group (Study 1)*

|  | | | | |  | | ***Group*** | | | | | | |  |  |
| --- | --- | --- | --- | --- | --- | --- | --- | --- | --- | --- | --- | --- | --- | --- | --- |
|  | | | | |  | | **TAU+MT**  **(n=30)** | | | | | | **TAU**  **(n=33)** |  |  |
| Age in years – M (SD) | | | | | |  | 43 (15) | | 41 (16) | | | | |  |  |
| Sex – N(%) | | |  | Male | | | | 2 (6.7) | | 3 (9.1) | | | |  |  |
|  | | |  | Female | | | | 28 (93.3) | | | | 29 (87.9) | |  |  |
|  | | |  | Other | | | | 0 (0.0) | | 1 (3.0) | | | |  |  |
| Race – N(%) |  | | White/Caucasian | | | | | 27 (90.0) | | | | 28 (84.8) | | |  |
|  |  | | American Native or White | | | | | 1 (3.3) | | | 0 (0.0) | | | |  |
|  |  | | White and Black | | | | | 0 (0.0) | | | 2 (6.0) | | | |  |
|  |  | | Asian | | | | | 0 (0.0) | | | 1 (3.0) | | | |  |
|  |  | | Black/African American | | | | | 1 (3.3) | | | 1 (3.0) | | | |  |
|  |  | | Hispanic^a^, American Native or White | | | | | 1 (3.3) | | | 1 (3.0) | | | |  |
| Education - N(%) | | High School Grad or equivalent | | | | | | 0 (0.0) | | 1 (3.0) | | | | | |
|  | | Some College or Technical School | | | | | | 7 (23.3) | | 5 (15.2) | | | | | |
|  | | Associate’s Degree | | | | | | 2 (6.7) | | 3 (9.1) | | | | | |
|  | | Bachelor’s Degree | | | | | | 7 (23.3) | | 16 (48.5) | | | | | |
|  | | Postgraduate Degree^b^ | | | | | | 14 (46.7) | | 8 (24.2) | | | | | |

*Notes*. Except for age which displays M(SD) for participants in each group,

number of participants are shown with % of the group’s sample size in parentheses.

^a^Includes Latinx or Spanish origin

^b^ Includes Master’s degree (MBA or equivalent) and doctorate

TAU + MT : Treatment as Usual + Mindfulness Training

| *Table S2. Study 1 one-way ANOVAs on Questionnaire Item z-Scores with CLUSTER as a Between-Subjects Factor* | | | | | | |
| --- | --- | --- | --- | --- | --- | --- |
|  | *F* | *P* | $\eta$*^2^* | Cluster 1 | Cluster 2 | Cluster 3 |
|  |  |  |  | n = 13 | n = 21 | n = 29 |
| *Questionnaire Item* | | | | M (SE) | M (SE) | M (SE) |
| PSWQ_1 ^a^ | 3.625 | 0.033* | 0.108 | 0.63(0.07) | -0.09(0.23) | -0.22(0.20) |
| PSWQ_2 | 4.698 | 0.013* | 0.135 | 0.72(0.11) | -0.17(0.25) | -0.20(0.18) |
| PSWQ_3 | 0.492 | 0.614 | 0.016 | 0.23(0.14) | -0.12(0.28) | -0.01(0.19) |
| PSWQ_4 | 4.857 | 0.011* | 0.139 | 0.66(0.14) | 0.03(0.24) | -0.32(0.19) |
| PSWQ_5 | 0.584 | 0.561 | 0.019 | 0.27(0.23) | -0.08(0.28) | -0.06(0.17) |
| PSWQ_6^b^ | 2.612 | 0.082 | 0.08 | 0.52(0.08) | -0.25(0.28) | -0.05(0.18) |
| PSWQ_7 ^b^ | 5.62 | 0.006* | 0.158 | 0.77(0.12) | -0.16(0.23) | -0.23(0.19) |
| PSWQ_8 | 0.572 | 0.567 | 0.019 | 0.23(0.38) | 0.03(0.25) | -0.13(0.14) |
| PSWQ_9 | 3.788 | 0.028* | 0.112 | 0.63(0.17) | -0.06(0.25) | -0.24(0.18) |
| PSWQ_10 | 2.008 | 0.143 | 0.063 | 0.23(0.00) | 0.23(0.00) | -0.27(0.27) |
| PSWQ_11 | 0.631 | 0.535 | 0.021 | -0.16(0.39) | 0.19(0.15) | -0.07(0.19) |
| PSWQ_12 | 0.255 | 0.775 | 0.008 | -0.14(0.33) | 0.11(0.22) | -0.02(0.18) |
| PSWQ_13 ^b^ | 2.812 | 0.068 | 0.086 | 0.56(0.22) | -0.09(0.25) | -0.19(0.17) |
| PSWQ_14 ^b^ | 5.158 | 0.009* | 0.147 | 0.63(0.30) | 0.10(0.20) | -0.36(0.17) |
| PSWQ_15 ^b^ | 10.434 | <0.001* | 0.258 | 0.88(0.21) | 0.07(0.22) | -0.45(0.16) |
| PSWQ_16 | 3.733 | 0.030* | 0.111 | 0.50(0.18) | 0.14(0.25) | -0.33(0.18) |
| GAD7_1 | 6.935 | 0.002* | 0.188 | 0.72(0.18) | 0.11(0.23) | -0.40(0.18) |
| GAD7_2 ^b^ | 9.511 | <0.001* | 0.241 | 0.88(0.18) | 0.02(0.25) | -0.41(0.15) |
| GAD7_3 ^b^ | 14.374 | <0.001* | 0.324 | 0.97(0.09) | 0.11(0.23) | -0.51(0.16) |
| GAD7_4 | 7.918 | 0.001* | 0.209 | 0.59(0.26) | 0.30(0.22) | -0.48(0.16) |
| GAD7_5 | 2.752 | 0.072 | 0.084 | 0.51(0.29) | 0.03(0.22) | -0.25(0.18) |
| GAD7_6 | 2.897 | 0.063 | 0.088 | 0.46(0.31) | 0.12(0.19) | -0.29(0.19) |
| GAD7_7 | 5.21 | 0.008* | 0.148 | 0.74(0.25) | -0.25(0.23) | -0.15(0.17) |
| FFMQ_NR_1 | 3.178 | 0.049* | 0.096 | -0.38(0.31) | -0.21(0.18) | 0.33(0.19) |
| FFMQ_NR_2 | 8.16 | 0.001* | 0.214 | -0.73(0.17) | -0.17(0.23) | 0.45(0.17) |
| FFMQ_NR_3 | 2.997 | 0.057 | 0.091 | -0.46(0.38) | -0.13(0.19) | 0.30(0.17) |
| FFMQ_NR_4 | 3.772 | 0.029* | 0.112 | -0.43(0.34) | -0.22(0.18) | 0.35(0.18) |
| FFMQ_NR_5 ^b^ | 4.182 | 0.020* | 0.122 | -0.50(0.26) | -0.18(0.23) | 0.35(0.17) |
| FFMQ_NR_6 | 2.375 | 0.102 | 0.073 | -0.51(0.25) | 0.05(0.25) | 0.19(0.17) |
| FFMQ_NR_7 ^b^ | 4.682 | 0.013* | 0.135 | -0.63(0.18) | -0.07(0.19) | 0.33(0.21) |
| MAIA_1_N | 13.315 | <0.001* | 0.307 | 0.25(0.28) | -0.77(0.19) | 0.44(0.15) |
| MAIA_2_N | 6.868 | 0.002* | 0.186 | 0.33(0.20) | -0.61(0.29) | 0.29(0.12) |
| MAIA_3_N | 4.735 | 0.012* | 0.136 | 0.12(0.37) | -0.51(0.19) | 0.32(0.16) |
| MAIA_4_N | 11.609 | <0.001* | 0.279 | 0.42(0.25) | -0.74(0.21) | 0.35(0.15) |
| MAIA_5_ND | 3.41 | 0.040* | 0.102 | -0.47(0.27) | -0.15(0.23) | 0.32(0.17) |
| MAIA_6_ND | 0.864 | 0.427 | 0.028 | -0.14(0.29) | 0.23(0.25) | -0.11(0.17) |
| MAIA_7_ND | 0.117 | 0.889 | 0.004 | -0.11(0.32) | 0.00(0.23) | 0.05(0.18) |
| MAIA_8_NW | 0.248 | 0.781 | 0.008 | -0.17(0.32) | 0.01(0.20) | 0.07(0.20) |
| MAIA_9_NW | 0.654 | 0.524 | 0.021 | -0.25(0.30) | 0.15(0.22) | 0.00(0.19) |
| MAIA_10_NW | 0.243 | 0.785 | 0.008 | 0.10(0.31) | -0.12(0.23) | 0.04(0.18) |
| MAIA_11_AR | 6.856 | 0.002* | 0.186 | 0.18(0.32) | -0.60(0.16) | 0.35(0.18) |
| MAIA_12_AR | 13.185 | <0.001* | 0.305 | -0.15(0.30) | -0.68(0.14) | 0.56(0.17) |
| MAIA_13_AR | 14.226 | <0.001* | 0.322 | -0.13(0.25) | -0.70(0.17) | 0.57(0.17) |
| MAIA_14_AR^b^ | 28.293 | <0.001* | 0.485 | 0.14(0.33) | -0.94(0.10) | 0.62(0.12) |
| MAIA_15_AR^b^ | 19.468 | <0.001* | 0.394 | 0.18(0.26) | -0.86(0.15) | 0.54(0.15) |
| MAIA_16_AR | 9.639 | <0.001* | 0.243 | 0.06(0.32) | -0.66(0.16) | 0.45(0.17) |
| MAIA_17_AR^b^ | 14.293 | <0.001* | 0.323 | 0.55(0.29) | -0.79(0.17) | 0.32(0.15) |
| MAIA_18_EA | 10.701 | <0.001* | 0.263 | 0.20(0.28) | -0.71(0.18) | 0.42(0.16) |
| MAIA_19_EA | 9.266 | <0.001* | 0.236 | 0.34(0.20) | -0.68(0.24) | 0.34(0.16) |
| MAIA_20_EA^b^ | 10.676 | <0.001* | 0.262 | 0.20(0.28) | -0.71(0.24) | 0.42(0.12) |
| MAIA_21_EA^b^ | 14.488 | <0.001* | 0.326 | 0.32(0.19) | -0.80(0.23) | 0.43(0.14) |
| MAIA_22_EA^b^ | 13.996 | <0.001* | 0.318 | 0.25(0.22) | -0.78(0.24) | 0.46(0.13) |
| MAIA_23_SR^b^ | 26.567 | <0.001* | 0.47 | -0.34(0.18) | -0.78(0.15) | 0.72(0.16) |
| MAIA_24_SR^b^ | 22.426 | <0.001* | 0.428 | 0.32(0.27) | -0.91(0.13) | 0.52(0.15) |
| MAIA_25_SR^b^ | 28.797 | <0.001* | 0.49 | 0.41(0.22) | -0.98(0.18) | 0.52(0.13) |
| MAIA_26_SR^b^ | 16.5 | <0.001* | 0.355 | 0.30(0.25) | -0.83(0.20) | 0.47(0.14) |
| MAIA_27_BL | 12.198 | <0.001* | 0.289 | 0.15(0.33) | -0.73(0.14) | 0.47(0.16) |
| MAIA_28_BL | 6.899 | 0.002* | 0.187 | 0.04(0.34) | -0.58(0.20) | 0.40(0.15) |
| MAIA_29_BL | 7.468 | 0.001* | 0.199 | -0.17(0.30) | -0.53(0.18) | 0.46(0.17) |
| MAIA_30_T | 6.849 | 0.002* | 0.186 | -0.62(0.22) | -0.22(0.21) | 0.44(0.18) |
| MAIA_31_T | 5.845 | 0.005* | 0.163 | -0.62(0.28) | -0.16(0.21) | 0.40(0.17) |
| MAIA_32_T | 8.992 | <0.001* | 0.231 | -0.41(0.25) | -0.46(0.18) | 0.52(0.18) |

**p* < 0.05, one-way ANOVAs.

^a^ Numbers to the right of questionnaire abbreviations refer to individual questionnaire items.

PSWQ: Penn State Worry Questionnaire; GAD-7: Generalized Anxiety Disorder 7-item Scale; FFMQ_NR: Non-Reactivity Subscale of the Five Facet Mindfulness Questionnaire.

MAIA_N: Noticing Subscale of the Multidimensional Assessment of Interoceptive Awareness (MAIA); MAIA_ND: MAIA ‘Not’-distract Subscale; MAIA_NW: MAIA ‘Not-worry Subscale; MAIA_AR: MAIA Attention Regulation Subscale; MAIA_EA: MAIA Emotional Awareness Subscale; MAIA_SR: MAIA Self-regulation Subscale; MAIA_BL: MAIA Body Listening Subscale; MAIA_T: MAIA Trusting Subscale.

^b^ Items retained following feature reduction

| *Table S3. Study 2 one-way ANOVAs on Questionnaire Item z-Scores with CLUSTER as a Between-Subjects*  *Factor* | | | | | | | |
| --- | --- | --- | --- | --- | --- | --- | --- |
|  | *F* | *P* | $\eta$*^2^* | Cluster 1 | Cluster 2 | Cluster 3 |  |
|  |  |  |  | n = 5629 | n = 2982 | n = 5399 |  |
| *Questionnaire Item* | | | | M (SE) | M (SE) | M (SE) |  |
| PSWQ_1^a^ | 109.773 | <0.001* | 0.015 | 0.07(0.01) | 0.15(0.02) | -0.15(0.01) |  |
| PSWQ_2 | 3901.646 | <0.001* | 0.358 | 0.73(0.01) | -0.45(0.01) | -0.51(0.01) |  |
| PSWQ_3 | 98.284 | <0.001* | 0.014 | 0.07(0.01) | 0.14(0.02) | -0.14(0.01) |  |
| PSWQ_4 | 4566.091 | <0.001* | 0.395 | 0.77(0.01) | -0.45(0.01) | -0.55(0.01) |  |
| PSWQ_5 | 4919.274 | <0.001* | 0.413 | 0.78(0.01) | -0.51(0.01) | -0.54(0.01) |  |
| PSWQ_6^b^ | 5221.104 | <0.001* | 0.427 | 0.80(0.01) | -0.49(0.01) | -0.56(0.01) |  |
| PSWQ_7 ^b^ | 6410.32 | <0.001* | 0.478 | 0.84(0.01) | -0.50(0.01) | -0.60(0.01) |  |
| PSWQ_8 | 631.669 | <0.001* | 0.083 | 0.26(0.01) | 0.16(0.02) | -0.36(0.01) |  |
| PSWQ_9 | 4737.808 | <0.001* | 0.404 | 0.77(0.01) | -0.49(0.01) | -0.54(0.01) |  |
| PSWQ_10 | 608.959 | <0.001* | 0.08 | 0.31(0.01) | 0.00(0.02) | -0.32(0.01) |  |
| PSWQ_11 | 657.465 | <0.001* | 0.086 | 0.25(0.01) | 0.20(0.02) | -0.37(0.01) |  |
| PSWQ_12 | 4303.625 | <0.001* | 0.381 | 0.75(0.01) | -0.49(0.01) | -0.52(0.01) |  |
| PSWQ_13 ^b^ | 5576.144 | <0.001* | 0.443 | 0.81(0.01) | -0.55(0.01) | -0.55(0.01) |  |
| PSWQ_14 ^b^ | 6623.477 | <0.001* | 0.486 | 0.85(0.01) | -0.50(0.01) | -0.61(0.01) |  |
| PSWQ_15 ^b^ | 7565.914 | <0.001* | 0.519 | 0.88(0.01) | -0.49(0.01) | -0.64(0.01) |  |
| PSWQ_16 | 4153.586 | <0.001* | 0.372 | 0.74(0.01) | -0.56(0.01) | -0.47(0.01) |  |
| GAD7_1 | 6072.7 | <0.001* | 0.464 | 0.83(0.01) | -0.49(0.01) | -0.59(0.01) |  |
| GAD7_2 ^b^ | 6305.73 | <0.001* | 0.474 | 0.84(0.01) | -0.49(0.01) | -0.60(0.01) |  |
| GAD7_3 ^b^ | 6944.694 | <0.001* | 0.498 | 0.86(0.01) | -0.52(0.01) | -0.61(0.01) |  |
| GAD7_4 | 5652.328 | <0.001* | 0.447 | 0.81(0.01) | -0.49(0.01) | -0.58(0.01) |  |
| GAD7_5 | 4526.818 | <0.001* | 0.393 | 0.76(0.01) | -0.46(0.01) | -0.54(0.01) |  |
| GAD7_6 | 4658.034 | <0.001* | 0.399 | 0.77(0.01) | -0.45(0.01) | -0.55(0.01) |  |
| GAD7_7 | 4762.321 | <0.001* | 0.405 | 0.77(0.01) | -0.45(0.01) | -0.56(0.01) |  |
| FFMQ_NR_1 | 296.26 | <0.001* | 0.041 | 0.01(0.01) | -0.36(0.02) | 0.18(0.01) |  |
| FFMQ_NR_2 | 759.839 | <0.001* | 0.098 | -0.19(0.01) | -0.34(0.02) | 0.39(0.01) |  |
| FFMQ_NR_3 | 967.64 | <0.001* | 0.121 | -0.19(0.01) | -0.41(0.02) | 0.43(0.01) |  |
| FFMQ_NR_4 | 1259.819 | <0.001* | 0.152 | -0.28(0.01) | -0.37(0.02) | 0.49(0.01) |  |
| FFMQ_NR_5 ^b^ | 1465.142 | <0.001* | 0.173 | -0.37(0.01) | -0.25(0.02) | 0.52(0.01) |  |
| FFMQ_NR_6 | 1378.183 | <0.001* | 0.164 | -0.33(0.01) | -0.31(0.02) | 0.51(0.01) |  |
| FFMQ_NR_7 ^b^ | 1753.598 | <0.001* | 0.200 | -0.42(0.01) | -0.22(0.02) | 0.56(0.01) |  |
| MAIA_1_N | 1582.933 | <0.001* | 0.184 | 0.32(0.01) | -0.81(0.02) | 0.12(0.01) |  |
| MAIA_2_N | 2189.748 | <0.001* | 0.238 | 0.39(0.01) | -0.90(0.02) | 0.09(0.01) |  |
| MAIA_3_N | 1632.067 | <0.001* | 0.189 | 0.06(0.01) | -0.79(0.02) | 0.38(0.01) |  |
| MAIA_4_N | 1911.555 | <0.001* | 0.214 | 0.31(0.01) | -0.88(0.02) | 0.17(0.01) |  |
| MAIA_5_ND | 898.524 | <0.001* | 0.114 | -0.37(0.01) | 0.50(0.01) | 0.12(0.01) |  |
| MAIA_6_ND | 933.483 | <0.001* | 0.118 | -0.14(0.01) | 0.66(0.02) | -0.22(0.01) |  |
| MAIA_7_ND | 1264.715 | <0.001* | 0.153 | -0.19(0.01) | 0.75(0.02) | -0.22(0.01) |  |
| MAIA_8_NW | 2050.629 | <0.001* | 0.226 | -0.57(0.01) | 0.52(0.01) | 0.31(0.01) |  |
| MAIA_9_NW | 2125.56 | <0.001* | 0.233 | -0.57(0.01) | 0.58(0.01) | 0.28(0.01) |  |
| MAIA_10_NW | 1005.84 | <0.001* | 0.126 | -0.01(0.01) | -0.61(0.01) | 0.34(0.01) |  |
| MAIA_11_AR | 1911.282 | <0.001* | 0.214 | -0.09(0.01) | -0.74(0.01) | 0.50(0.01) |  |
| MAIA_12_AR | 2545.282 | <0.001* | 0.267 | -0.08(0.01) | -0.84(0.01) | 0.54(0.01) |  |
| MAIA_13_AR | 2132.046 | <0.001* | 0.233 | -0.11(0.01) | -0.75(0.01) | 0.53(0.01) |  |
| MAIA_14_AR^b^ | 2730.007 | <0.001* | 0.28 | -0.11(0.01) | -0.83(0.01) | 0.58(0.01) |  |
| MAIA_15_AR^b^ | 2717.267 | <0.001* | 0.28 | -0.13(0.01) | -0.82(0.01) | 0.58(0.01) |  |
| MAIA_16_AR | 2407.059 | <0.001* | 0.256 | -0.12(0.01) | -0.79(0.02) | 0.56(0.01) |  |
| MAIA_17_AR^b^ | 2994.707 | <0.001* | 0.3 | -0.15(0.01) | -0.83(0.02) | 0.61(0.01) |  |
| MAIA_18_EA | 2416.536 | <0.001* | 0.257 | 0.18(0.01) | -0.96(0.02) | 0.34(0.01) |  |
| MAIA_19_EA | 2567.789 | <0.001* | 0.268 | 0.39(0.01) | -0.97(0.02) | 0.13(0.01) |  |
| MAIA_20_EA^b^ | 2746.929 | <0.001* | 0.282 | 0.11(0.01) | -0.98(0.02) | 0.43(0.01) |  |
| MAIA_21_EA^b^ | 2886.025 | <0.001* | 0.292 | 0.08(0.01) | -0.99(0.02) | 0.46(0.01) |  |
| MAIA_22_EA^b^ | 2858.057 | <0.001* | 0.29 | 0.10(0.01) | -0.99(0.02) | 0.45(0.01) |  |
| MAIA_23_SR^b^ | 2812.504 | <0.001* | 0.287 | -0.31(0.01) | -0.61(0.02) | 0.66(0.01) |  |
| MAIA_24_SR^b^ | 3091.824 | <0.001* | 0.306 | -0.23(0.01) | -0.75(0.02) | 0.66(0.01) |  |
| MAIA_25_SR^b^ | 2839.012 | <0.001* | 0.288 | -0.23(0.01) | -0.72(0.02) | 0.64(0.01) |  |
| MAIA_26_SR^b^ | 2874.502 | <0.001* | 0.291 | -0.25(0.01) | -0.70(0.02) | 0.65(0.01) |  |
| MAIA_27_BL | 2297.659 | <0.001* | 0.247 | -0.10(0.01) | -0.79(0.01) | 0.54(0.01) |  |
| MAIA_28_BL | 1759.496 | <0.001* | 0.201 | -0.16(0.01) | -0.64(0.01) | 0.52(0.01) |  |
| MAIA_29_BL | 1995.217 | <0.001* | 0.222 | -0.13(0.01) | -0.71(0.01) | 0.53(0.01) |  |
| MAIA_30_T | 2260.652 | <0.001* | 0.244 | -0.25(0.01) | -0.62(0.02) | 0.60(0.01) |  |
| MAIA_31_T | 2405.059 | <0.001* | 0.256 | -0.27(0.01) | -0.61(0.02) | 0.62(0.01) |  |
| MAIA_32_T | 2299.936 | <0.001* | 0.247 | -0.18(0.01) | -0.71(0.02) | 0.58(0.01) |  |

**p* < 0.001, one-way ANOVAs.

^a^ Numbers to the right of questionnaire abbreviations refer to individual questionnaire items
PSWQ: Penn State Worry Questionnaire

GAD-7: Generalized Anxiety Disorder 7-item Scale

FFMQ_NR: ‘Non-Reactivity’ Subscale of the Five Facet Mindfulness Questionnaire

MAIA_N: Noticing Subscale of the Multidimensional Assessment of Interoceptive Awareness (MAIA)

MAIA_ND: MAIA ‘Not’-distract Subscale

MAIA_NW: MAIA ‘Not-worry Subscale

MAIA_AR: MAIA Attention Regulation Subscale

MAIA_EA: MAIA Emotional Awareness Subscale

MAIA_SR: MAIA Self-regulation Subscale

MAIA_BL: MAIA Body Listening Subscale

MAIA_T: MAIA Trusting Subscale

^b^ Items retained following feature reduction


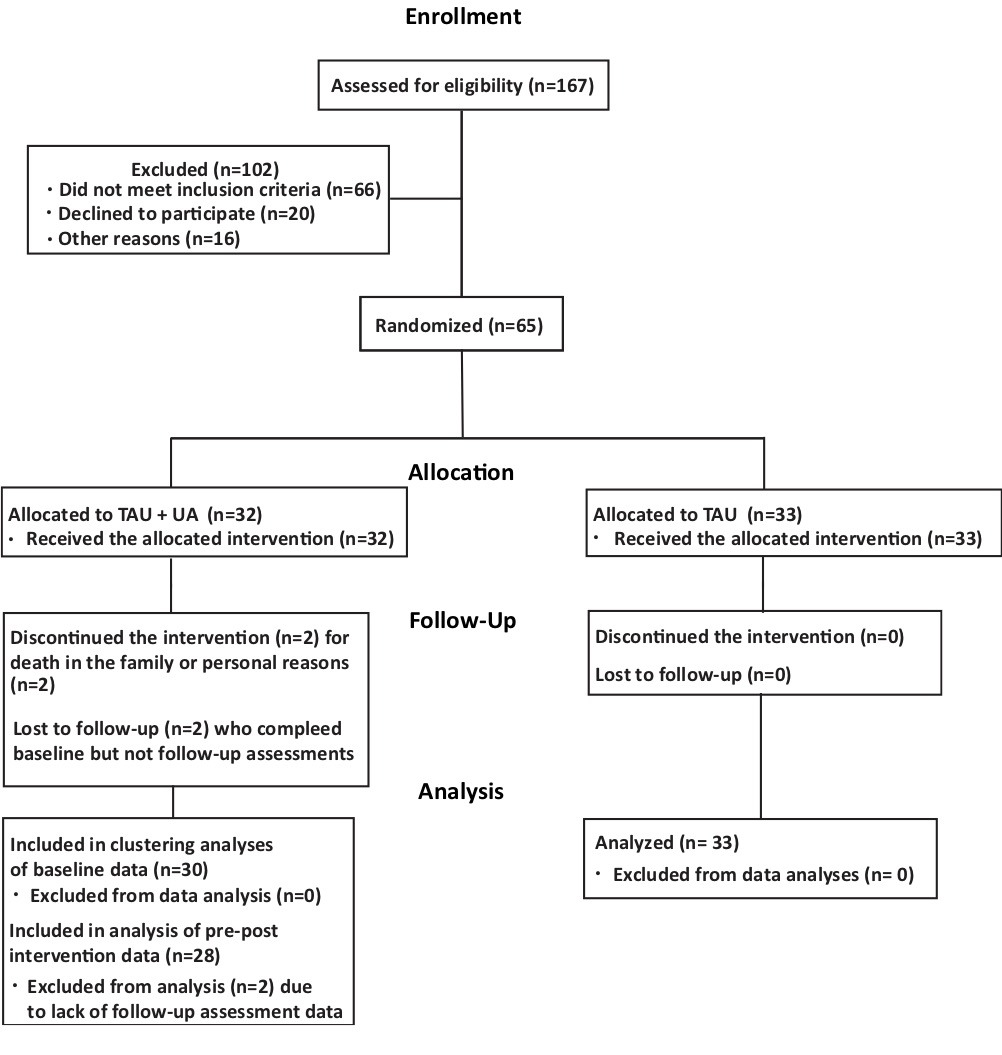


**MT**

**completed**

*Figure S1.* Study 1 CONSORT diagram illustrating the participant flow process. Adapted from Roy and colleagues (2021) [2].

TAU + MT: Treatment as Usual + Mindfulness Training
